# Screening Mold Colonies by Using Two Toxicity Assays Revealed Indoor Strains of *Aspergillus calidoustus* Producing Ophiobolins G and K

**DOI:** 10.3390/toxins11120683

**Published:** 2019-11-21

**Authors:** Marja Johanna Salo, Tamás Marik, Ottó Bencsik, Raimo Mikkola, László Kredics, András Szekeres, Maria A. Andersson, Heidi Salonen, Jarek Kurnitski

**Affiliations:** 1 Department of Civil Engineering, Aalto University, Box 12100, FI-00076 Aalto, Finland; raimo.mikkola@aalto.fi (R.M.); aino.andersson@aalto.fi (M.A.A.); heidi.salonen@aalto.fi (H.S.); jarek.kurnitski@aalto.fi (J.K.); 2 Department of Microbiology, Faculty of Science and Informatics, University of Szeged, Közép fasor 52, 6727 Szeged, Hungary; mariktamas88@gmail.com (T.M.); bencsikotto@gmail.com (O.B.); laszlo.kredics@gmail.com (L.K.); andras.j.szekeres@gmail.com (A.S.); 3 Department of Civil Engineering and Architecture, Tallinn University of Technology, Ehitajate tee 5, 19086 Tallinn, Estonia

**Keywords:** *Aspergillus calidoustus*, ophiobolins, indoor mold, fluorescence

## Abstract

The occurrence and toxin production of the opportunistic pathogen *Aspergillus calidoustus* in Finnish buildings is not well documented in the literature. We tracked and identified four *A. calidoustus* colonies cultivated from indoor settled dusts and revealed the biological activities of crude biomass extracts. The toxic substances were identified as 6-epi-ophiobolin K, ophiobolin K, and ophiobolin G by high-performance liquid chromatography–mass spectrometry (HPLC-MS) based on chromatographic and mass spectrometry data (MS and MS/MS) on the crude extract of *A. calidoustus* strain MH34. A total of 29 fungal colonies collected from settled dust in an office room reported for indoor-air-related illnesses were screened for toxins that inhibited boar sperm motility in the BSMI (boar sperm motility inhibiting) assay and cell proliferation in the ICP (inhibition of cell proliferation) assays with PK-15 cells. Out of the 27 colonies tested as toxic, 12 colonies exhibiting conidiophores representative of the genera *Chaetomium*, *Penicillium*, *and Paecilomyces* were excluded from the study, while 13 colonies exhibited *Aspergillus*-like conidiophores. Biomass suspensions of these colonies were divided into two categories: Category 1 colonies (n = 4), toxic in the BSMI assay and the ICP assays, emitted blue fluorescence and grew at 37 °C; Category 2 colonies (n = 9), only toxic in the ICP assay, emitted orange fluorescence and exhibited limited or no growth at 37 °C. Colonies in Category 1 were pure-cultured, and the strains were named as MH4, MH21, MH34, MH36. Strain MH34 was identified as *A. calidoustus* by the internal transcribed spacer (ITS) sequences. Ethanol-soluble dry substances extracted from the biomass of the pure cultures exhibited a toxicological profile in the BSMI assay, SMID (sperm membrane integrity damage) assay, and ICP assay similar to that exhibited by pure ophiobolin A. Overall, the viable conidia of *A. calidoustus* in indoor settled dusts deserve attention when potentially hazardous mold species are monitored.

## 1. Introduction

Representatives of *Aspergillus* section *Usti* are commonly reported in built environments in Europe and North America [1,2]. A previously documented polyphasic taxonomic approach, including molecular data, based on the internal transcribed spacer (ITS), calmodulin and β-tubulin sequences, extrolite profiling, morphological criteria, and growth at 37 °C revealed that section *Usti* consists of 21 species [3]. Members of section *Usti* produce extrolites unique to the genus *Aspergillus*, including ophiobolins known to be produced by *Aspergillus calidoustus* [2].

*A. calidoustus* differs from *A. ustus* lat. *ustus* in its ability to grow at 37 °C and it being an opportunistic pathogen [2,3]. *A. calidoustus* strains have already been isolated from various environments, like indoor air in Germany, wooden construction materials in Finland as well as clinical and indoor samples from the Netherlands, Norwegian drinking water distribution systems, and built environments in Canada [2,3,4]. Moreover, building-derived strains of *Aspergillus* section *Usti*, probably mostly *A. calidoustus* strains, isolated from chipboard and gypsum liner were suggested as producers of ophiobolin-type compounds [5,6]. Clinical strains of *A. calidoustus* from Europe have been shown to produce ophiobolin G and H, and ophiobolin production has been suggested as a virulence factor [2]. *A. calidoustus* is considered a relatively rare human pathogen primarily causing cutaneous aspergillosis and eye infections but is also associated with high mortality rates due to its resistance to antifungal drugs [7].

*A. calidoustus* is therefore a novel, toxin-producing potential pathogen detected in indoor environments, and requires attention as an emerging hazardous indoor contaminant.

Secondary metabolites ophiobolins are sesterterpenoids produced by filamentous fungi belonging to the genera *Bipolaris, Drechslera*, *Cephalosporium*, *Ulocladium*, and *Aspergillus*. Most of the fungal secondary metabolites can help the fungus occupy its growth substratum as well as survive in natural environments, but they may also have toxic effects on humans and animals [8,9]. The toxic effects of ophiobolins in vitro and low acute toxicity in in vivo experiments have been extensively studied due to the suspected anticancerous activity of ophiobolins [9]. Additionally, ophiobolin A, 6-epi-ophiobolin K, and ophiobolin K pure compounds displayed in previous studies antifungal activity, antiprotozoal activity, and cytotoxic and mitochondriotoxic activity in human tumoral and continuous cell lines [10,11]. Ophiobolin A-induced calmodulin inhibitory effect and growth-inhibitory effects in mammalian cells and slowly proliferating mammalian cells are less sensitive to growth-inhibitory effects of ophiobolin A than their highly proliferative counterparts [10,11].

The in vivo toxicity of ophiobolins is not well documented in the literature, and ophiobolin-induced intoxications in humans have not ever been reported; however, the rapid release of inflammatory mediators and systemic inflammation promoted by exposure to ophiobolin A [12] and mitochondrial dysfunctions [7,8], recorded after prolonged exposure to ophiobolins. Ophiobolin family with many structurally similar analogues [13] could cause health effects in exposed humans.

Indoor strains of *A. calidoustus* isolated from Canadian buildings did not produce ophiobolins under the conditions tested by Slack et al. [2,3]. The prevalence of ophiobolin producers among indoor *A. calidoustus* strains, the indoor occurrence of this potential pathogen, and the identity of the different ophiobolins produced by indoor strains are currently unknown. Furthermore, ophiobolins produced by indoor *Aspergillus* strains and their toxic activities have gained little attention in the field as indoor hazards.

This study describes ophiobolin-producing indoor *A. calidoustus* strains isolated from settled indoor dusts from an office room in a 1960s university building in Finland. It also describes a novel battery of methods enabling the tracking of ophiobolin-producing *A. calidoustus* strains and their separation from other *Aspergillus* strains growing similarly from the same dust samples. Furthermore, the biological activities of the crude biomass extracts containing ophiobolins are revealed in a battery of bioassays as well as the HPLC-MS-based identification of these compounds.

## 2. Results

### 2.1. Screening Single Colonies for Toxicity in Two Bioassays Divided Yellow–White Aspergillus Colonies in Two Categories Differing in Toxic Responses and Fluorescence Emission

**Figure 1:**
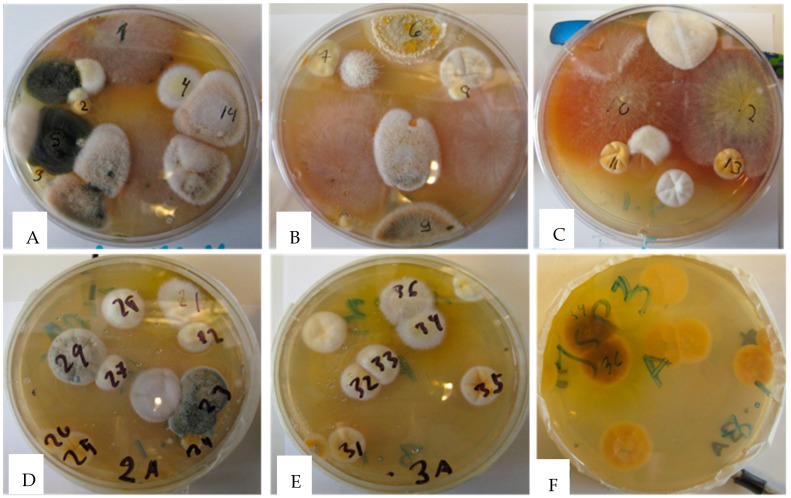
Indoor fungi grown for three weeks on malt extract agar from a swab wiped from an office in a university building in Finland. The office was abandoned by the occupant due to serious indoor air-related illness. Each of the numbered colonies on Panels (**A**–**E**) were separately tested for the presence of toxins. The reverse side of the plate in Panel (**E**) is shown in Panel (**F**) with the dark reverse of colonies 34 and 36 in contrast to colonies 31, 32, and 33.

Mold colonies (n = 29) cultivated from settled indoor dust on malt extract plates (Figure 1) were screened for toxicity via two bioassays. The colonies exhibited five responses based on toxicity of the assays and were divided into five categories: Category (1) colonies toxic in both assays, cytostatic to PK-15 cells (ICP, inhibition of cell proliferation assay), and inhibiting sperm motility after exposure for 30 min (BSMI, boar sperm motility inhibition assay); Category (2) colonies toxic only in the ICP assay, nontoxic to sperm cells; Category (3) colonies toxic in both assays but need one day for toxic response in the BSMI assay; Category (4) colonies toxic only in the BSMI assay after 30 min and/or one day of exposure, not cytostatic to the PK-15 cells in the ICP assay; and Category (5) colonies provoking no responses in the toxicity assays.

**Figure 2:**
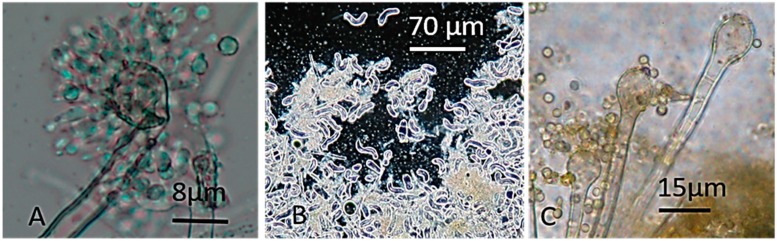
Micrographs showing the morphology of the *Aspergillus* conidiophores exhibited by colonies in Categories 1 and 2. Panels (**A**,**B**) show the conidiophore and Hülle cells of colony 34 and represent the morphology of colonies in Category 1 toxic to both sperm cells and PK-15 cells. Panel (**C**) shows the conidiophores of colony 31 and represents the colonies in Category 2, toxic only to PK-15 cells.

The most toxic colonies representing Category 1, numbers 4, 21, 34, and 36 in Figure 1, were toxic in both the ICP and BSMI assays. Biomass suspensions of the colonies completely inhibited sperm motility and the proliferation of porcine kidney cells (PK-15 cells) after 30 min and 24 h of exposure, respectively. The biomass suspensions contained hyphae, conidia, and liquid vesicles picked from the surface of the plate-grown colonies shown in Figure 1. The four colonies were white and yellow with a dark reverse side (Figure 1) and exhibited the same *Aspergillus*-like morphology, which is represented by colony No. 34 in Figure 2A–C, and all four colonies grew at 37 °C.

The colonies in Category 2, the nine colonies numbered 10, 11, 12, 13, 25, 26, 32, 33, and 35 in Figure 1, which were also found to exhibit *Aspergillus*-like conidiophores (Figure 2), were very toxic in the ICP assay, provoked no response in the BSMI assay, and exhibited no or very weak growth at 37 °C. These colonies were also yellow or white, but their reverse side was pale (Figure 1). Colonies in Categories 3 to 5, exhibiting the morphology of conidiophores indicative of the genera *Chaetomium*, *Penicillium*, *Paecilomyces*, and *Cladosporium*, were excluded from this study.

**Figure 3:**
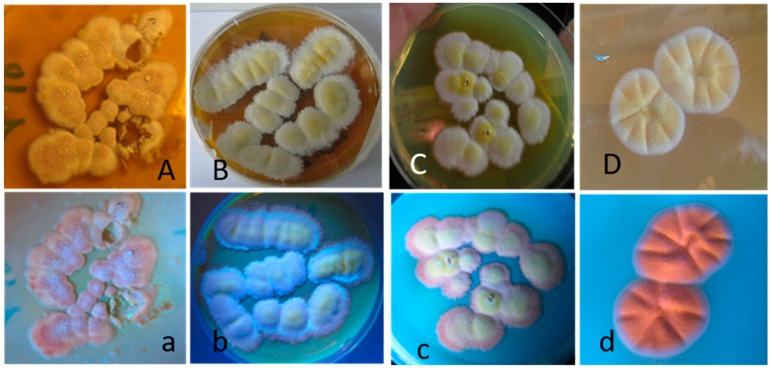
Pure cultures of *Aspergillus*-like strains pictured in visible light (upper row) and UV light (lower row). The cultures were grown on malt extract agar at 22 °C for 10 days. The picture shows the white–yellow colonies of the strains MH4, MH21, MH34 in Panels (**A**–**C**) (uppercase), respectively. The same colonies emitting violet–blue fluorescence are shown in Panels (**a**–**c**) (lowercase). Panels (**D**) (uppercase) and (**d**) (lowercase) show the white–yellow colonies of strain MH10 in visible light and the colonies emitting orange fluorescence in UV light, respectively.

The 13 yellow–white *Aspergillus*-like colonies in Categories 1 and 2 were pure-cultured on malt extract agar, named MH4, MH21, MH34, MH36 and MH10, MH13, MH25, MH26, MH32, MH33, MH35, and pictured in visible and UV light (360 nm). Representatives of the pure cultures are shown in Figure 3. All *Aspergillus*-like pure cultures were light white–yellow in visible light but differed in color in the fluorescence emissions. When excited with UV light, pure cultures in Category 1 (Figure 3A–C) emitted violet-blue emissions (Figure 3a–c), whereas pure cultures in Category 2 emitted strong orange fluorescence, as represented by MH10 in Figure 3D and Figure 3d.

**Figure 4:**
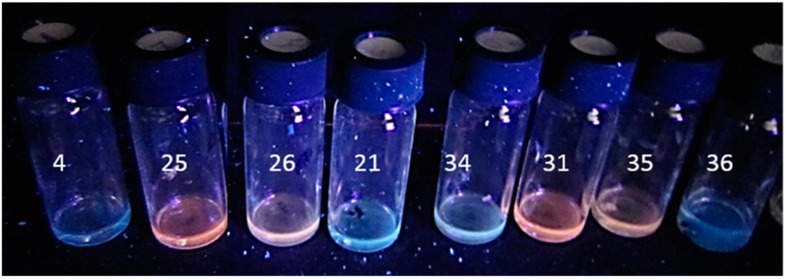
Biomass suspensions of single mold colonies photographed when excited with UV light (360 nm). The colonies toxic in both assays are Colonies 4, 21, 34, and 36, which emitted blue fluorescence. Suspensions of the colonies toxic only to PK-15 cells emitted orange fluorescence (Colonies 25, 26, 31, and 35).

To test if fluorescence emission would speed up and confirm the division of the *Aspergillus* cultures into two categories, the biomass suspensions of the single colonies as well as the pure cultures were inspected for fluorescence emissions when excited with UV light (360 nm). Biomass suspensions of the single colonies (No. 4, 21, 34, and 36 in Figure 4) as well as the pure cultures obtained from them (MH4, MH21, MH34, and MH36) (data not shown) emitted blue fluorescence, whereas the colonies in Category 2 (25, 26, 31, and 35 in Figure 4), toxic only to PK-15 cells in the ICP assay, emitted orange fluorescence. Biomass suspensions of the pure cultures, reference strain, *A. versicolor* SL/3, and pure sterigmatocystin emitted similar orange fluorescence (data not shown). Based on fluorescence emission and responses to the toxicity assays, the *Aspergillus*-like colonies and biomass suspensions were divided into the blue-fluorescence-emitting colonies, which were toxic in both the BSMI and ICP assays, and the orange-fluorescence-emitting colonies, which were toxic only in the ICP assay against the PK-15 cells.

### 2.2. The Four Blue-Fluorescing Strains Exhibited a Uniform Toxicity Profile in Four Bioassays and Were Identified as Aspergillus calidoustus

Toxic compounds from the biomass of pure cultures (grown on malt extract agar) of the 13 *Aspergillus*-like strains were extracted with ethanol. The toxicological profiles of the ethanol-soluble dry substances were investigated via a set of four toxicity assays. Selected *Aspergillus*, *Trichoderma*, *Chaetomium*, and *Bipolaris* strains from our previous strain collection were investigated for toxic responses and mycotoxin production, and were used as reference strains. The toxic responses of the crude extracts of the reference strains were compared to the responses obtained from commercially available pure mycotoxins. The toxic responses in the four bioassays, expressed as EC_50_ concentrations of the dry substances in the crude extracts and of the pure mycotoxins, are summarized in Table 1.

Strain MH34, considered similar to strains MH4, MH21, and MH36 based on the toxicity pattern shown in Table 1, fluorescence, morphology of its colonies and conidiophores (Figure 1, Figure 2, Figure 3 and Figure 4), and its ability to grow at 37 °C, was identified as *A. calidoustus* based on ITS sequence analysis. Extracts from the *Aspergillus calidoustus* strains MH4, MH21, and MH36, and from the ophiobolin A producing *Bipolaris oryze* SZMC 13003 as well as pure ophiobolin A were characterized by high toxicity (0.5–2 µg mL^−1^) in the BSMI assay after one day of exposure, a 10-fold decrease in toxic endpoint if the exposure of the sperm cells was prolonged from one hour to one to two days, and high toxicity (0.5–1 µg mL^−1^) in the ICP assay. The other reference strains exhibited high toxicity in the BSMI compared to the toxicity in the ICP assay (*T. atroviride* Tri335, *A. westerdijkiae* PP2, *Chaetomium globosum* HS1) or high toxicity in the ICP assay compared to the toxicity in the BSMI assay (*A. versicolor* SL/3).

The results also indicated that the crude extracts of the *A. calidoustus* strains may have contained substances with bioactivities similar to those of ophiobolin A. These substances expressed cytostatic activity in proliferating somatic cells and motility-inhibiting toxicity in exposed sperm cells at similar low concentrations to that of ophiobolin A. The motility-inhibiting activity recorded with the BSMI assay for the *A. calidoustus* extracts was caused by a sublethal effect, since the EC_50_ concentrations of the motility inhibition in the BSMI assay was 10 times lower than the EC_50_ concentrations in the SMID assay measuring lethal toxicity as a loss of plasma membrane integrity.

Crude extracts of the orange-fluorescence-emitting *Aspergillus* sp. strains MH10, MH12, MH25, MH26, MH32, MH33, and MH35 and the reference strain *A. versicolor* SL/3 provoked no response in the sperm assay (BSMI assay) after short-term exposure (≤1 h), but the extracts and pure sterigmatocystin were highly toxic to proliferating PK-15 cells in the ICP assay. These results indicated that the extracts from the blue-fluorescing *A. calidoustus* strains contained different toxic substances than the extracts from the nine orange-fluorescing *Aspergillus* sp. strains.

### 2.3. Toxic Compounds in the Crude Extract of Aspergillus calidoustus Were Identified as Ophiobolins

The toxic compounds from *A. calidoustus* strain MH 34 provoking toxic response in the BSMI and ICP assays were identified using a bioactivity-guided approach, that is, bioassay-directed high-performance liquid chromatography (HPLC) fractionation which resulted in the isolation of the toxic compounds. The bioassays identified 2 HPLC fractions out of 26 fractions tested, which inhibited sperm motility and proliferation of PK-15 cells, respectively. Toxic compounds of *A. calidoustus* strain MH 34 were identified using high-performance liquid chromatography–mass spectrometry (HPLC-MS). Compound **1** at retention time 4.2 min had a protonated mass ion [M+H]^+^ at *m*/*z* 385.5 and a sodiated mass ion [M+Na]^+^ at *m*/*z* 407.5 and dimer [2M+Na]^+^ at *m*/*z* 791.4. The MS/MS spectra of the precursor mass ion at *m*/*z* 791.4 produced a sodiated mass ion at *m*/*z* 407.5, indicating that *m*/*z* 791.4 mass ion was [2M+Na]^+^ dimer. The MS/MS spectra of the precursor mass ion at *m*/*z* 385.5 gave similar *m*/*z* 201, 307, 349 mass fragment ions as the published reference MS/MS spectra of the mass ion *m*/*z* 385 of 6-epi-ophiobolin K [17]. Compound **2** at retention time 4.2 min had a protonated mass ion [M+H]^+^ at *m*/*z* 385.5, a sodiated mass ion [M+Na]^+^ at *m*/*z* 407.5, and [2M+Na]^+^ dimer at *m*/*z* 791.4. The MS/MS spectra of the precursor ion at *m*/*z* 791.4 produced a sodiated mass ion at *m*/*z* 407.5, indicating that *m*/*z* 791.4 mass ion was [2M+Na]^+^ dimer. The MS/MS spectra of the precursor mass ion at *m*/*z* 385.5 gave similar *m*/*z* 109, 201, 239, 331, 349 mass fragment ions as the published reference MS/MS spectra of the mass ion *m*/*z* 385 of ophiobolin K [13]. Compound **3** at retention time 5.1 min had a protonated mass ion [M+H]^+^ at *m*/*z* 367.2 and a sodiated mass ion [M+Na]^+^ at *m*/*z* 389.2 and [2M+H]^+^ and [2M+Na]^+^ dimers at *m*/*z* 733.0 and 756.0, respectively. The MS/MS spectra of the precursor mass ions at *m*/*z* 733.0 and 756.0 produced protonated mass ions at *m*/*z* 367.2 and sodiated *m*/*z* 389.2, indicating that mass ions at *m*/*z* 733.0 and 756.0 were [2M+H]^+^ and [2M+Na]^+^ dimers, respectively. The MS/MS spectra of the precursor mass ion at *m*/*z* 367.2 gave similar *m*/*z* 293.1, 307.2, 331.3, 349.2 mass fragment ions as the published reference MS/MS spectra of the corresponding mass ions of ophiobolins [13]. Based on retention times, MS and MS/MS mass spectrometric analysis, and the published mass spectrometric data of ophiobolins [13], compounds **1**, **2**, and **3** were identified as 6-epi-ophiobolin K, ophiobolin K, and ophiobolin G, respectively.

### 2.4. The Crude Extract of Aspergillus calidoustus Strain MH34 Provoked Sublethal Toxic Effects in Exposed Sperm Cells and Somatic Cells

**Figure 5:**
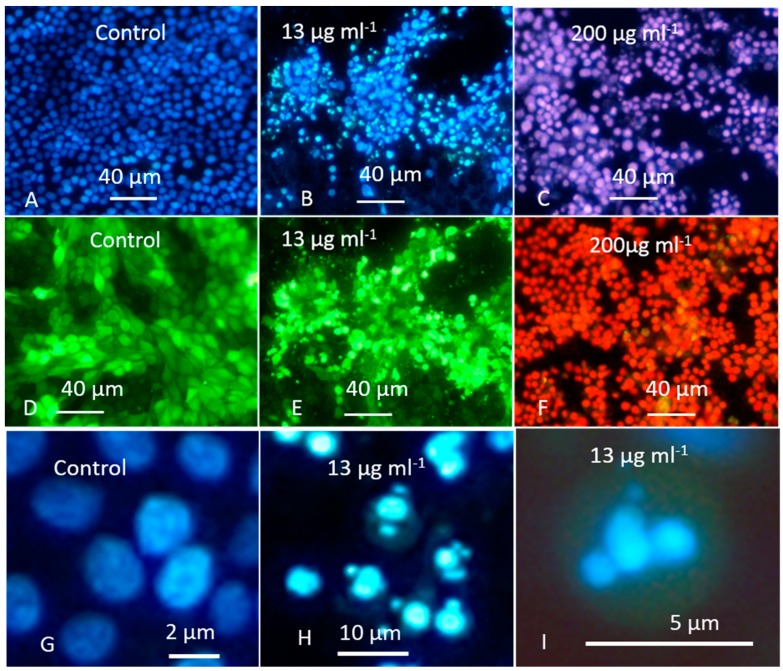
Fluorescence micrographs of porcine kidney cells (PK-15) exposed as resting cells in the monolayer to crude extracts (13 µg mL^−1^ and 200 µg mL^−1^) from *A. calidoustus* MH34. The cells were stained with the triple-stain Hoechst 33342 + calcein-AM + propidium iodide. The DNA stain Hoechst 33342 stains nuclei in both living and dead cells and emits blue fluorescence. Calcein-AM is cleaved by intracellular esterases to calcein, emitting green fluorescence in live cells with intact plasma membranes. The DNA stain propidium iodide permeates only dead cells, and nuclei in cells that have lost their plasma membrane integrity emit purple or red fluorescence. Panels in the first and third rows were inspected using filters to help see blue/purple emissions of Hoechst 33342 and propidium iodide (no green emission of calcein was visible), whereas the filters used for the panels in the second row helped to see green/red emissions of calcein-AM and propidium iodide, respectively (no blue emission was visible). The control cells in Panels (**A**) and (**G**) exposed to the ethanol solvent only exhibited blue fluorescence in intact nuclei in cells with plasma membranes impermeable to propidium iodide. The intactness of the plasma membrane integrity is indicated by the absence of purple emission in Panels (**A**) and (**G**) and confirmed by the green emission of the control cells in Panel (**D**). Cells in Panel (**B**) exposed to the crude extract at concentrations of 13 µg mL^−1^ exhibited fragmentation of blue nuclei in c. 50% of the cells, and a lack of purple emission indicated the intactness of the plasma membrane, confirmed by the green emission of the same cells pictured using the green/red filters, as shown in Panel (**E**). Details of the fragmented nuclei and nuclear fragments in cells with intact plasma membranes are shown in Panels (**H**) and (**I**) (no purple emissions visible). Panels (**C**) and (**F**) show cells exposed to 200 µg mL^−1^ of the crude extract. The nuclei in 100% of the plasma-membrane-damaged necrotic cells stained with propidium iodide emitted purple and red fluorescence (no blue or green emissions visible).

The crude extract of *A. calidoustus* strain MH34 containing 6-epi-ophiobolin K, ophiobolin K, and ophiobolin G provoked fragmented nuclei in resting PK-15 cells. Cells exposed to the solvent only are shown in Figure 5A,D,G. Fragmented nuclei visible in c. 50% of the cells (Figure 5B) with intact, blebbing plasma membranes (Figure 5E) were visible in fluorescence micrographs after exposure to concentrations of 13–26 µg mL^−1^. Detailed pictures of fragmented nuclei and nuclear fragments in cells with intact plasma membranes are shown in Figure 5H,I, respectively. Necrotic cell death, visible as purple and red emissions in 100% of cells permeable to propidium iodide, occurred at exposure concentrations of 125 to 250 µg mL^−1^ (Figure 5C,F). These results indicate that sublethal toxicity, visible as fragmentation of nuclei in PK-15 cells with intact plasma membranes, occurred at 10-fold smaller concentrations than the lethal toxicity indicated by necrotic cell death.

**Figure 6:**
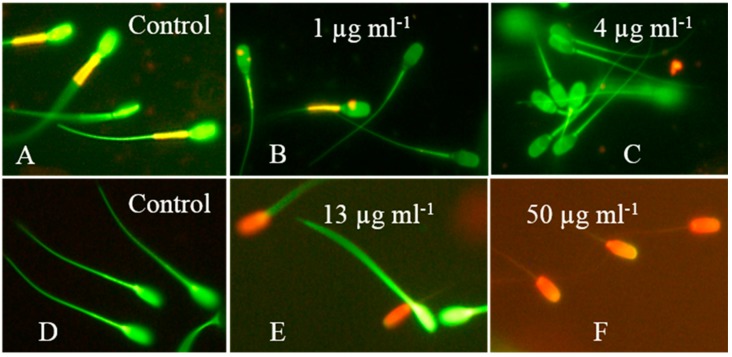
Epifluorescence micrographs of boar sperm exposed to crude extract from *A. calidoustus* strain MH34. The sperm cells in the upper row were stained with the potentiometric dye JC-1, which stains membranes with high membrane potential ΔΨ orange and membranes with low ΔΨ green. Sperm cells in the lower row were stained with the viability stain calcein-AM + propidium iodide. Cells with intact plasma membranes are stained green, and cells with depleted plasma membrane integrity are stained red. Panel (**A**) shows sperm cells exposed to the solvent only exhibiting high ΔΨ in the mitochondrial sheath located in the midpiece of the sperm cells. Panels (**B**,**C**) show the concentrations for which 50% and 100% of sperm cells, respectively, exhibited depolarized mitochondria, as indicated by a decrease in orange emission from the mitochondrial sheath. Panel (**D**) shows control-exposed sperm cells with intact plasma membranes emitting green fluorescence. Panels (**E**,**F**) show sperm cells exposed to concentrations causing 50% and 100% depletion of the plasma membrane integrity, respectively.

The sublethal effects of crude extracts from *A. calidoustus* MH34 on exposed boar sperm are shown in Figure 6. Sperm cells stained with the potentiometric dye JC-1 emit orange fluorescence in the mitochondrial sheath if the membrane potential ΔΨ is high (≥−140 mV). Green fluorescence emitted by the mitochondrial sheath indicates low membrane potential (≤−100 mV) in sperm cells with intact plasma membranes. Active mitochondria in the mitochondrial sheath in the neck of the sperm cells emitted orange fluorescence, as shown in the control panel in Figure 6A. The mitochondrial sheath in sperm cells exposed to 1–4 µg mL^−1^ of the crude extract emitted green fluorescence, as shown in Figure 6B,C. The plasma membranes of these sperm cells were intact and impermeable to propidium iodide, which indicated the depletion of the mitochondrial ΔΨ. Figure 6 shows that the sublethal toxicity indicated by the depolarization of the mitochondria occurred at 10-fold lower concentrations than the lethal toxicity, indicated by the loss in plasma membrane integrity (Figure 6D–F). Thus, the sublethal toxic effects recorded for the crude extract of *A. calidoustus* MH34, depletion of mitochondrial ΔΨ, and inhibition of motility occurred at the same concentrations (Figure 6 and Table 1).

## 3. Discussion

This study first describes 6-epi-ophiobolin K-, ophiobolin K-, and ophiobolin G-producing *A. calidoustus* strains isolated from an indoor environment in Finland. Indoor *A. calidoustus* strains have also been isolated in Canada, but, instead of ophiobolins, these strains were found to produce a novel sesquiterpene, drimane, and two isoquinoline alkaloids [2]. Additionally, infection-related *A. calidoustus* strains have been shown to produce ophiobolin G and H in Europe [2].

This study described a battery of simple, rapid, and cost-effective methods for tracking the diversity of mycotoxin-producing indoor molds, focusing on the genus *Aspergillus.* The methods included: (a) screening biomass suspensions of single colonies for toxic responses using two toxicity assays measuring different toxic mechanisms; (b) revealing the fluorescence of biomass suspensions when excited with UV light; (c) revealing growth at 37 °C; and (d) assigning the colony to the genus *Aspergillus* based on the morphology of the conidiophores.

The two screening assays detected sublethal toxins disturbing cellular energy metabolism (BSMI) as well as toxins affecting cellular proliferation (ICP) [18]. Screening of the biomass suspensions from individual colonies separated the ophiobolin-producing colonies by exhibiting a low toxic response (EC_50_ < 2 µg mL^−1^) in both toxicity assays. The same results were obtained for ophiobolin A by Bencsik et al. [16]. The toxic response and emission of blue fluorescence separated the *Aspergillus calidoustus* colonies from the numerically dominant *Aspergillus* colonies growing on the same plate. These colonies were toxic only in one assay and emitted orange fluorescence.

The approach of using toxicity screening has been instrumental in tracking toxigenic species (especially new species producing novel toxins) from indoor environments and foods. *Acremonium exuviarum* produces the novel mitochondrial toxin acrebol [19]. Amylosin-producing *Bacillus amyloliquefaciens* from the indoor environment, paenilid-producing *Paenibacillus tundrae*, and antimycin-producing Streptomycetes from cereals were also found by toxicity assays [20,21,22]. In this approach, toxicity assays were used not to stress the health hazard connected to the occurrence of certain microbes in a building but for revealing the diversity of toxigenic indoor microbes by categorizing microbial colonies prior to final identification.

The toxic effects of the 6-epi-ophiobolin K-, ophiobolin K-, and ophiobolin G-containing crude extracts on exposed sperm cells and PK-15 cells were found to be similar to the toxic effects of the purified ophiobolin A. In other words, motility inhibition, mitochondrial depolarization, and cytostatic activity occurred at similar concentrations, whereas plasma membrane damage occurred at 10-fold higher concentrations [16]. Fragmentation of nuclei was detected in resting PK-15 cells after the exposure of the monolayer to 6-epi-ophiobolin K-, ophiobolin K-, and ophiobolin G-containing crude extract from *A. calidoustus* MH34, which has also been described in relation to ophiobolin A in the L1210 cell line. An apoptosis-like cell death process was also observed in *M. circinelloides* after 1.6 μg mL^−1^ ophiobolin A treatment and for ophiobolin O in MCF-7 cells [23,24,25,26]. Ophiobolin A was able to induce paraptosis-like cell death in human glioblastoma multiforme cells by decreasing the big/large conductance Ca^2+^-activated K^+^ channel activity [8,9]. The K^+^ channel activity could explain the mitochondria-depolarizing and motility-inhibiting effects recorded for the boar sperm cells.

Exposure to ophiobolin A was shown to promote the rapid release of inflammatory mediators and promote systemic inflammation [12]. Infection-related *A. calidoustus* strains have been shown to produce ophiobolin G [2]. Moreover, the ability to produce ophiobolin G may contribute to the virulence of infectious *A. calidoustus* strains [2]. It was hypothesized that viable ophiobolin-producing *Aspergillus calidoustus* colonies in the indoor environment may represent a threat to human health because: a) exposure to ophiobolin emitted by indoor strains could modulate immune responses and contribute to symptoms experienced in moldy buildings and b) ophiobolin-producing strains could be virulent pathogens.

## 4. Conclusions

This study described a bioactivity-guided, bioassay-directed screening procedure for tracking the diversity of cultivated indoor *Aspergillus* colonies and enabling rapid recognition of *Aspergillus calidoustus* colonies producing ophiobolins. The results indicated that crude extracts containing 6-epi-ophiobolin K, ophiobolin K, and ophiobolin G, crude extracts containing ophiobolin A, and pure ophiobolin A seem to have similarities in their biological activities. The occurrence of potentially pathogenic and toxin-producing *A. calidoustus* strains could also be related to indoor air quality.

## 5. Materials and Methods

### 5.1. Experimental Design

Mold colonies were cultivated from settled dust and collected 1.5 to 2 m above the ground level at a university office, in which the occupants complained about indoor air-related problems. A total of 29 colonies displaying unique colony morphology in daylight were screened for toxicity using two complementary rapid screening tests. The tests measured (a) the toxins affecting cellular energy metabolism, mitochondria, and ion homeostasis as the inhibition of motility of boar spermatozoa (BSMI) [18,19,27] and (b) toxins affecting macromolecular synthesis and cytostatic activity as the inhibition of proliferation of a somatic cell line, PK-15 (ICP) [16]. The toxicity tests performed with the colonies from the primary isolation plates (Figure 1) were repeated with pure, single-spore cultures. The most toxic colonies (n = 4) and the numerically dominant toxic colonies (n = 9) were pure-cultured into single-spored fungal strains and their biomasses extracted for ethanol-soluble substances. The toxicity profiles in the four bioassays measuring sublethal, lethal, and cytostatic toxicity were elucidated. The assays measured sublethal toxicity in exposing resting boar sperm as BSMI after exposure for 30 min (rapid inhibition) and one day (slow inhibition), lethal toxicity as sperm membrane integrity damage (SMID), and cytostatic toxicity as ICP of PK-15 cells. The test protocols for the BSMI and SMID assays exposing the resting boar sperm cells are described in [17,18] and in [17] for the ICP assay. A randomly chosen colony, MH34, of the four most toxic colonies was identified at the species level, and the toxicity profiles were compared with the ethanol extracts of the selected indoor molds, commercial mycotoxins, and mycotoxins purified by the present authors. Toxic substances were purified with HPLC and identified by LC-MS.

### 5.2. Cultivation of Mold Colonies

The settled dust samples, c. 10 mg, were seeded on plates containing a malt extract medium (15 g malt extract from Sharlab, Barcelona, Spain and 12 g of agar from Amresco, Dallas, USA, in 500 mL of H_2_O) or a tryptic soy extract agar (Sharlab, Barcelona, Spain, 20 g L^−1^ in 500 mL) medium without antibiotics or fungicides and were sealed at the site of sampling with gas-permeable adhesive tape. Colonies were inspected and counted after one, two, and three weeks of culturing at 23 ± 2 °C. After three weeks of incubation, the colonies on the primary isolation plates (not yet single-spored) were numbered and screened for toxicity.

The ability to grow at 37 °C was tested on malt extract agar plates sealed with gas-permeable tape, which were then incubated for five days. The strains *Trichoderma longibrachiatum* THG and *T. atroviride* H1/226 were used as positive and negative controls, respectively [17]

### 5.3. Rapid Screening Tests with Ex Vivo and In Vitro Assays

Rapid screening tests applied directly to the primary sampling plates where the dust had been cultivated were designed for mycotoxin-producing indoor molds. Settled dust samples were cultivated on malt extract agar without added antibiotics. The plates were sealed with gas-permeable tape and incubated for four weeks at room temperature. The biomass of 10 to 20 mg of the fungal colonies was dispersed in 0.2 mL ethanol in a sealed glass vial and heated in a water bath of 80 °C for 10 min. The obtained biomass dispersals were used for exposing boar spermatozoa (BSMI assay) and porcine kidney cells PK-15 (ICP assay) in ex vivo and in vitro bioassays, respectively. A colony was considered exhibiting sublethal toxicity in the BSMI assay if ≤2.5 mg mL^−1^ of the biomass inhibited sperm motility after 30 min and/or one day of exposure. Cytostatic toxicity was indicated if ≤5 mg mL^−1^ of the biomass inhibited the cell proliferation (ICP assay) of the PK-15 cells exposed for one to two days [13]. The toxicity assays performed with ethanol extract from pure fungal cultures were performed using porcine cells (sperms, somatic cell lines) as indicators according to [14,15,16,19,20].

### 5.4. Toxicity Assays for Ethanol-Soluble Dry Substances Extracted from a Plate-Grown Fungal Biomass and Pure Mycotoxins

The plate-grown biomass of the fungal pure cultures (100–300 mg) was extracted with ethanol, as described in [18]. The toxicity assays, the BSMI (boar sperm motility inhibition) assay after exposures of 30 min (rapid inhibition) and one day (slow inhibition), SMID (sperm membrane integrity damage) assay, and ICP (inhibition of cell proliferation) assay are all described in [17,18].

Cell death recorded in PK-15 cells grown as monolayers for 48 h was measured after 24 h of toxin exposure as the permeability to propidium iodide and the inhibition of glucose consumption according to [11,12].

### 5.5. Calculation of EC_50_, the Half Maximal Effective Concentrations for the Ethanol Dry Substances and Pure Mycotoxins in the BSMI, SMID, and ICP Assays

In the BSMI assay, the EC_50_ concentration for motility inhibition was concluded to be the toxin concentration closest to that provoking a >50% decrease in the number of sperm cells exhibiting rapid tail beating (compared to the sperm cells in the solvent control as described in [16,17]). Rapid tail beating is visible under the microscope as an artefact; sperm cells with rapidly beating tails look like sperm cells having two tails, and these two-tailed cells may be counted and their proportion of the total number of sperm cells estimated [16].

The EC_50_ was calculated from the equation of the straight line between EC_50-40_ and EC_80-90_: Y = DY/DX 9 X + C where Y is the motility closest to 50% of the motility of the solvent control, X is the EC_50_ concentration, and C is a constant between 100% and 60%. All tests were run in triplicate and differences between the replicate tests were within one dilution step (twofold). The sperm assays were calibrated with triclosan and valinomycin.

In the SMID assay, the EC_50_ (half maximal effective concentration) corresponded to the concentration causing a 50% decrease in mortality compared to the positive dead control (=100% mortality). The lower the EC_50_ value, the more toxic the substance. The assay was calibrated with triclosan in three parallel tests, and the EC_50_ was 2 µg mL^−1^ (SD ± 30%). In the SMID assay, mortality (permeability to PI) in the exposed sperm cells samples was calculated using the following equation [17]:

$$\mathrm{Loss}\;\mathrm{of}\;\mathrm{viability}\;\mathrm{of}\;\mathrm{sample}\;(\%)=\frac{\mathrm{Fluorescence}\;\mathrm{of}\;\mathrm{sample}\;-\;\mathrm{background}}{\mathrm{Fluorescence}\;\mathrm{of}\;\mathrm{dead}\;\mathrm{control}\;-\;\mathrm{background}}\times 100$$

In the ICP assay, the EC_50_ was calculated as follows as described in [13]: The inhibition of cell proliferation was inspected with a phase contrast microscope. EC_100_ for the inhibition of proliferation was easy to observe due to the absence of intact cells in the well. EC_0_ was also easy to observe as there was an intact cell monolayer indistinguishable from the control. With the microscope, determining EC_50_ was more difficult. To achieve a more exact value for EC_50_, the inhibition ability of resazurin reduction was investigated. Resazurin is a nontoxic, cell-permeable, redox-sensitive phenoxazine [28]. Therefore, after treatment, the plates were incubated for three days at 37 °C, 10 μL resazurin (Sigma Chemical Co., St. Louis, MO, USA) (400 μg mL^−1^ in normal saline) was added to the wells, and the plates were incubated again for two hours under the same conditions. Then, the plates were analyzed by a microtiter plate reader (Fluoroskan Ascent, Thermo Scientific, Vantaa, Finland) at the excitation and emission wavelengths of 544 nm and 590 nm, respectively. The toxicity was expressed as the lowest concentrations in which the ratio of the living cells was less than 50% (EC_50_). The cell toxicity assays were repeated three times. This EC_50_ fitted between EC_100_ and EC_0_ observed with the microscope; the maximal difference between the two methods was one dilution step. The assay was calibrated with triclosan (Sigma Chemical Co., St. Louis, MO, USA), and the EC_50_ in the 10 parallel tests was 9.4 µg mL^−1^ (SD ± 3.4).

### 5.6. Fluorescence Microscopy

PK-15 cells grown into a monolayer for 48 h were exposed for 18 h to the crude extract of MH 34, and then triple-stained with the viability stains Hoechst 33342 + propidium iodide + calcein–AM, as described in [29,30,31] and inspected with a fluorescence microscope (Nikon Eclipse E600; Nikon Corporation, Tokyo Japan). The calcein-AM-stained cells with intact and/or blebbing plasma membranes exhibited green fluorescence, while the propidium-iodide-dyed cells with damaged plasma membranes exhibited red fluorescence after inspection with filters of 450 to 490 nm (excitation), band-pass and 520 nm emission, long-pass. The Hoechst-33342-stained, fragmented apoptotic nuclei were blue and the propidium-iodide-stained necrotic nuclei were red (dead) after inspection with filters of 330 to 380 (excitation) and 480 nm (emission).

### 5.7. Identification of the Fungal Strains

The reference strains *A. westerdijkiae* PP2 and *A. versicolor* SL/3 were identified at DSMZ (Deutche Samlung vor Mikroorganismen und Zellkulturen) in 2004 and 2008, respectively. *Aspergillus calidoustus* MH34, *Trichoderma atroviride* Tri335, and *Chaetomium globosum* HS5 were identified based on the amplification of the internal transcribed spacer (ITS1–5.8S rDNA–ITS2) region of the ribosomal RNA gene cluster with primers ITS1 (5′-TCCGTAGGTGAACCTGCGG-3′) and ITS4 (5′-TCCTCCGCTTATTGATATGC-3′) [32] based on [33]. The *A. calidoustus* MH34 ITS sequence is deposited in GenBank (KM853016.1). Although ITS sequence analysis alone is not able to discriminate between the species *A. calidoustus* and *A. pseudodeflectus*, the presence of Hülle cells clearly confirms the ITS-based diagnosis of strain MH34 as *A. calidoustus*, as *A. pseudodeflectus* is not forming any Hülle cells [33].

### 5.8. Purification of Ophiobolin A

The purification of ophiobolin A was carried out according to Bencsik et al. [13]. *Bipolaris oryzae* strain SZMC 13003 was cultivated in a potato dextrose broth (PDB) medium at 28 °C. After 12 days of cultivation, fungal cultures were filtered using a cheese cloth filter, and the fermented broth was extracted with an equal volume of ethyl acetate. The crude extract was fractionated by a semipreparative normal-phase high-performance liquid chromatography (HPLC) system using (ethylacetate) EtOAc/n-hexane 1:1 at a flow rate of 2 mL min^−1^. Further purification was carried out with two consecutive semipreparative RP-HPLC separations. The applied mobile phases were (water:acetonitrile) H_2_O:MeCN 3:7 (3 mL min^−1^) and H_2_O:MeCN 5:5 (3 mL min^−1^). During the purification procedure, the purity of ophiobolin A was tested using analytical HPLC measurements at a wavelength of 230 nm.

### 5.9. HPLC-MS Analysis and Identification of Mycotoxins in the Fungal Extracts

The biomass (400 mg ± 50 mg) of *A. calidoustus* MH34 was harvested from a malt extract agar plate grown at room temperature for 14 days. The fungal toxins were extracted from the collected biomass with ethanol, and the ethanolic extract was fractionated by high-performance liquid chromatography (HPLC). The columns used for the HPLC fractionation were reversed phase C18 column Atlantis T3 (100 Å, 3 µm, 4.6 mm × 150 mm), and for the HPLC-MS analysis, reversed phase C18 SunFire (100 Å, 2.5 μm, 2.1 mm × 50 mm) (Waters, Milford, MA, USA). The HPLC-electrospray ionization ion trap mass spectrometry analysis (ESI-IT-MS) was performed using an MSD-Trap-XCT plus ion trap mass spectrometer at a mass range of *m*/*z* 50–2000 equipped with an Agilent ESI source and Agilent 1100 series LC (Agilent Technologies, Wilmington, DE, USA). HPLC-MS analysis was performed using an isocratic elution. The eluents were 0.1% formic acid in water (A) and acetonitrile (B). The separation of the extract of *A. calidoustus* was performed using 75% B for 10 min at a flow rate of 0.2 mL min^−1^. The fractions were tested for toxicity as described in [34].

### 5.10. Reagents and Supplies

5,50,6,60-Tetrachloro-1,10,3,300-tetraethylbenzimidazolyl-carbocyanine iodide (JC-1, 3520-43-2, MW 65223), propidium iodide (PI, 25535-16-4, MW 66839), calcein-AM (148504-34-1, MW 99486), and Hoechst 33342 (23491-52-3, MW 61599) were from Invitrogen (Carlsbad, CA, USA). The purification of ophiobolin A, as well as stephacidin B and avrainvillamide, is described in [16] and [14], respectively. Alamethicin A4665 (*Trichoderma arundinaceum*) (27061-78-5, a mixture of alamethicin F50 peptaibols, MW 1962, 1976, 1976, and 1990), sterigmatocystin, and ochratoxin A were obtained from the Sigma-Aldrich Corporation (St. Louis, MO, USA). Malt extract and tryptic soy agar were from Scharlab (Barcelona, Spain). The other chemicals were of an analytical grade and purchased from local suppliers.

## Figures and Tables

**Table 1:** Toxic endpoints for the crude extracts of indoor *Aspergillus calidoustus* and *Aspergillus* sp. strains. The extracts were tested in twofold dilutions. The strains were compared to selected reference strains and selected pure reference mycotoxins in a set of toxicity assays exposing boar spermatozoa and porcine kidney PK-15 cells.

	EC_50_ µg mL^−1^					
	Boar Sperm			PK-15		
	BSMI ^1^		SMID ^2^	ICP ^2^		
	0.5–1 h	1–2 days	1 day	2 days		
*A. calidoustus* MH4	38	2	13	1		
*A. calidoustus* MH21	30	0.5	13	0.5		
*A. calidoustus* MH34	50	1	11	1		
*A. calidoustus* MH36	40	0.5	13	1		
*Aspergillus* sp. MH10	>100	10	>100	1		
*Aspergillus* sp. MH11	>100	20	>100	2		
*Aspergillus* sp. MH25	>100	20	>100	2		
*Aspergillus* sp. MH26	>100	20	>100	2		
*Aspergillus* sp. MH32	>100	10	>100	3		
*Aspergillus* sp. MH33	>100	20	>100	1		
*Aspergillus* sp. MH35	>100	20	>100	1		
Reference Strains					Mycotoxin Produced	References
*A. westerdijkiae* PP2	10	5	>50	15	Avrainvillamide Stephacidin B Ochratoxin A	[14]
*A. versicolor* SL/3	250	20	>100	1	Sterigmatocystin Averufin	[15]
*B. oryzae* SZMC 13003	>25	5		6	Ophiobolin A	[16]
*T. atroviride* Tri335	10	3	3	30	Trichorzianines	[15]
*C. globosum* HS1	10	5	>20	50	Chaetoglobosins	[17]
*T. reesei* DSM 768 ^3^	250	>50	>50	500	None	[17]
Reference Mycotoxins						References
Alamethicin	5	0.2	0.2	8		[17]
Avrainvillamide	0.6	0.3	>2	0.3		[14]
Ochratoxin A	>100	>50	>50	>50		[14]
Ophiobolin A	2.5	0.25	2	0.2		[16]
Stephacidin B	0.5	0.2	>2	0.2		[14]
Sterigmatocystin	>100	>20	>20	0.25		[14]

^1^ BSMI (boar sperm motility inhibition assay), the average difference between three measurements were within one dilution step. ^2^ SMID (sperm membrane integrity damage assay) and ICP (inhibition of cell proliferation assay), the SD between three measurements was < ± 35%. ^3^ Negative control representing the upper limit of the assay.
